# In Vitro Fecal Fermentation of *Euphorbia humifusa*-Derived Polysaccharides and Their Protective Effect against Ulcerative Colitis in Mice

**DOI:** 10.3390/foods12040751

**Published:** 2023-02-08

**Authors:** Ning Xiang, Jianbo Zhao, Siqiao Chang, Shasha Li, Shuwen Liu, Chan Wang

**Affiliations:** 1 Guangdong Provincial Key Laboratory of New Drug Screening, School of Pharmaceutical Sciences, Southern Medical University, 1838 Guangzhou Avenue North, Guangzhou 510515, China; 2 Division of Vascular and Interventional Radiology, Department of General Surgery, Nanfang Hospital, Southern Medical University, Guangzhou 510515, China; 3 State Key Laboratory of Organ Failure Research, Guangdong Provincial Institute of Nephrology, Southern Medical University, Guangzhou 510515, China

**Keywords:** *Euphorbia humifusa* polysaccharides, absorption, fermentation, ulcerative colitis

## Abstract

*Euphorbia humifusa* is a plant species with medicinal and food characteristics used to treat diarrhea and other intestinal diseases. This study investigated the prebiotic effects of *E. humifusa*-derived polysaccharides (EHPs) on human colonic microbiota and their regulatory effects on ulcerative colitis (UC). Structural characterization showed that EHPs mainly consisted of galactose, glucose, and glucuronic acid and were heteropolysaccharides having molecular weights of 7.70 × 10^3^ and 1.76 × 10^2^ kDa, respectively. EHPs were identified as poorly absorbed macromolecules, verified by the apparent permeability coefficient values (Papp < 1.0 × 10^−6^ cm/s) and cellular uptake by Caco-2 cell monolayers. During in vitro fermentation studies, the contents of acetic, propionic, and valeric acids increased significantly in EHP-supplemented samples after 24 h compared to that in the control sample. Moreover, EHPs could alter the intestinal microbiota composition by increasing the relative abundance of *Bifidobacterium* and *Holdemanella* and reducing that of *Escherichia-Shigella*, *Tyzzerella*, and *Parasutterella* at the genus level. In a dextran sulfate sodium (DSS)-induced UC mouse model, EHPs alleviated UC symptoms by increasing the colon length, reversing the colon tissue damage and inhibiting pro-inflammatory cytokines. Overall, these results suggest that EHPs could be utilized as a potential prebiotic or a promising nutritional strategy for UC management.

## 1. Introduction

Ulcerative colitis (UC) is a chronic and recurrent inflammatory bowel disease. Most UC lesions start appearing in the rectum and can spread throughout the colon, and the characteristic symptoms include abdominal pain, bloody stools, and weight loss [1,2]. Although UC pathogenesis is still not fully elucidated, several recent studies have indicated a relationship between intestinal microbiota and UC. The intestinal microbiota is an important frontier in understanding the development and progression of UC and other diseases [3]. Gut microbiota usually attaches to the intestinal mucosal layer and plays crucial functions in maintaining host health, such as regulating the body’s immunity, enhancing nutrient absorption, preserving the integrity of the intestinal barrier, and increasing pathogen defense ability [4]. In the past decade, accumulating studies have shown that manipulating the intestinal flora could alleviate various chronic diseases, such as inflammatory bowel disease, obesity, hypertension, diabetes, and liver disorders. Thus, increasing efforts have been made to explore microbiologically-guided interventions to improve human health [5,6]. 

**Figure 1:**
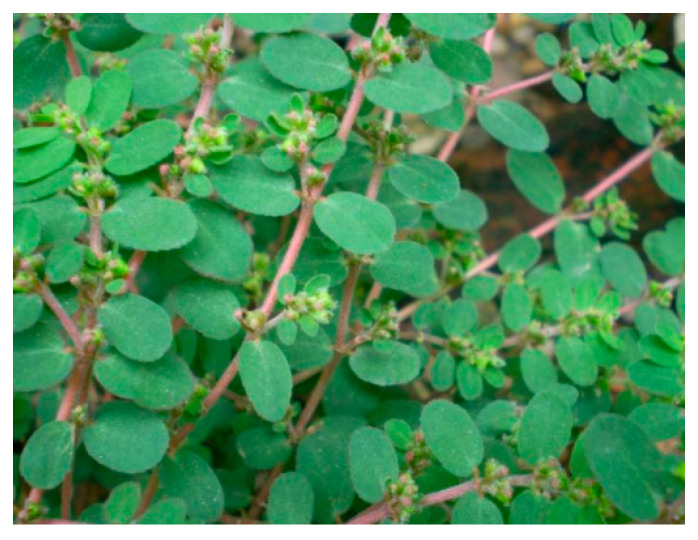
A picture of fresh *Euphorbia humifusa*.

Polysaccharides have attracted impressive attention as regulators of intestinal microbiota. Because of specific digestive enzymes lacking in the human body, most polysaccharides can pass through the gastrointestinal tract to the colon, where they can be fermented by intestinal microbiota [7,8]. Notably, the fermentation of polysaccharides exerts a positive effect against UC, which could be attributed to its influence on inflammatory cytokines and intestinal microbiota [9]. Additionally, metabolites produced during colonic fermentation, including short-chain fatty acids (SCFAs), could promote the growth of probiotic organisms and prevent intestinal disorders [3]. Exploring the potential health benefits of polysaccharides has become one of the hot issues in current functional food and medical research. The plant species *Euphorbia humifusa* is widely distributed across Eastern Asia and has been used as a folk medicine in China and a health assistance food in Korea [10,11] (Figure 1). According to the Chinese Pharmacopeia (2020 edition), *E. humifusa* has been utilized in the treatment of dysentery and enteritis, with a suggestive role in maintaining gut ecosystem stability [10,11]. Considering these findings, we speculated that *E. humifusa* might be applied in UC treatment via regulating intestinal microbiota.

The effect of natural plants on intestinal bacteria is firmly known to be related to their active polysaccharides; however, little information is presently available on *E. humifusa*-derived polysaccharides (EHPs). Therefore, this study aims to evaluate the potential therapeutic effects of EHPs on UC. Although most polysaccharides can transit into the large intestine with few physicochemical changes in the upper digestive tract of humans, the absorption mechanism of EHPs remains unclear. Firstly, we employed a Caco-2 cell monolayer model to investigate the absorption behavior of EHPs. Secondly, the effects of EHPs on intestinal microbiota composition and SCFAs were studied via in vitro fermentation. Lastly, the anti-inflammatory and beneficial effects of EHPs were evaluated in UC mouse models. These findings could provide an alternative therapy for UC and promote the development and utilization of EHPs.

## 2. Materials and Methods

### 2.1. Materials

*E. humifusa* was harvested from the Anguo traditional Chinese medicine market (Baoding, Hebei Province, China). All monosaccharide standards were purchased from Solarbio Science and Technology Co. (Beijing, China), and the dextran standard was procured from Aladin company (Guangzhou, Guangdong Province, China). Other reagents used were of analytical grade.

### 2.2. Extraction and Purification of EHPs

According to a previous method [12], 20 g of E. humifusa ultrafine powder was extracted with 800 mL deionized water at 95 °C for 2 h. The crude polysaccharides were then concentrated and precipitated using 80% ethanol (*v*/*v*), followed by centrifugation at 4000 rpm for 10 min, and the precipitation was dissolved in distilled water and deproteinized by the Sevag method. The protein-removed sample was dialyzed against distilled water for 24 h (8–14 kDa molecular mass cutoff) and precipitated again in 80% ethanol (*v*/*v*). After centrifugation, the precipitate was freeze-dried.

### 2.3. Structural Characterization of EHPs

#### 2.3.1. Chemical Composition

The total carbohydrate content of EHPs was determined by the phenol-sulfuric acid method [13], and the amount of protein was measured using Coomassie brilliant blue G250 [14].

#### 2.3.2. Molecular Weight (Mw) Distribution

The Mw of polysaccharides was determined using high-performance size exclusion chromatography, as described previously by Xia et al. [15]. The instrument was equipped with a multi-angle laser light scattering system (DAWN, HELEOS, Wyatt Technology Co., Santa Barbara, CA, USA), connected with a refractive index detector (Waters-2414, Milford, MA, USA) and the MALLS system (Waters 2695, Milford, MA, USA), equipped with columns of TSK-gel G-3000 PW XL (300 × 7.8 mm, Tokyo, Japan) and TSK-gel G5000PWXL columns (300 × 7.8 mm, Tokyo, Japan). Briefly, the EHP solution (1 mg/mL) was passed through a 0.22 μm filter, and 20 μL of the sample was injected into the system. The experimental conditions were as follows: the mobile phase consisted of a 0.9% NaCl solution at a flow rate of 0.5 mL/min and 35 °C. 

#### 2.3.3. Monosaccharide Composition

EHPs (5 mg) were hydrolyzed by trifluoroacetic acid (TFA, 3.0 M, 2 mL) at 120 °C for 4 h in an ampoule bottle. After removing excess TFA, the resulting products were derivatized with 1-phenyl-3-methyl-5-pyrazolone (PMP). The PMP-labeled samples were analyzed using high-performance liquid chromatography (HPLC, L-20A, Shimadzu, Japan) with an XDB-C18 chromatographic column (250 mm × 4.6 mm, 5 μm, Agilent Technologies, Santa Clara, CA, USA). Glucose (Glc), galactose (Gal), galacturonic acid (GalA), glucuronic acid (GlcA), rhamnose (Rha), arabinose (Ara), xylose (Xyl), and mannose (Man) standards were combined for use as a mixed standard. The mobile phase was the mixture of acetonitrile and phosphate buffer (17:83, *v*/*v*). Samples (20 μL) were applied to the HPLC system (1.0 mL/min, 25 °C), and detected at 250 nm by a UV detector [16].

#### 2.3.4. Fourier Transform Infrared (FTIR) Spectroscopic Analysis

The polysaccharide powder was mixed with spectroscopic potassium bromide (KBr) powder (200 mg), then pressed into pellets, and the spectra were recorded using an FTIR spectrometer (Thermo Fisher Scientific, Waltham, WA, USA) in the 400–4000 cm^−1^ region [17].

### 2.4. In Vitro Absorption of EHPs

#### 2.4.1. Fluorescent Labeling of Polysaccharides

To understand the absorption characteristics and cellular uptake of EHPs by the Caco-2 cell (American Type Culture Collection, ATCC, HTB037) monolayers, EHPs were covalently labeled with fluorescein isothiocyanate (FITC), named FITC-EHPs and the fluorescence substitution degree was calculated to be 1.76% by UV-visible spectroscopy [18].

#### 2.4.2. Caco-2 Cell-Based Intestinal Absorption Model

The cellular intestinal model was established based on a previously reported method [18]. Briefly, a 0.5 mL cell suspension (1 × 10^5^ cells/mL) was seeded into a 12-well polycarbonate transwell chamber and incubated at 37 °C in an atmosphere of 5% CO_2_ for 21 days to allow cell monolayer formation. The integrity of the Caco-2 cell monolayers was evaluated by measuring values of transepithelial electrical resistance (TEER). Only the cells with TEER values higher than 500 Ω·cm^2^ were selected for transport studies. Furthermore, we explored the transport of FITC-EHPs (200 μg/mL) from the apical to basolateral side across Caco-2 cell monolayers. The fluorescence absorption was measured to estimate the concentration of EHPs, and the apparent permeability coefficient (Papp) was also determined. The cytotoxicity of FITC-EHPs to Caco-2 cells was assayed using the cell counting kit-8 (CCK-8) assay (Beyotime, Shanghai, China). The Caco-2 cells were prepared and dispersed in 96-well cell culture plates at a cellular density of 1.0 × 10^5^ cells per well. After incubating with different concentrations of EHPs for 24 h, 10 μL of CCK-8 solution in PBS was added to each well and incubated at 37 °C for 2 h. In the end, the optical density of each well was measured by using a microplate reader at 450 nm.

#### 2.4.3. EHP Uptake by Caco-2 Cells

Following a previously reported method by Wang et al. [18], Caco-2 cells with a density of 1 × 10^5^/mL were seeded in a 48-well plate at 37 °C for 5 days, followed by incubation with a 1 mL solution of FITC-EHPs (1000 μg/mL) for 4 h. After incubation, the cellular uptake of EHP was observed by laser scanning confocal microscopy (LSCM, Olympus, Japan).

### 2.5. In Vitro Fermentation of EHPs 

#### 2.5.1. Fermentation

Fecal samples were provided by three healthy volunteers (age: 20–25) who had not taken antibiotics or probiotics in the past three months. All samples were mixed with phosphate buffer saline to obtain fecal suspensions (20%, *w*/*v*), which were then filtered through two layers of sterile gauze sponges and immediately transferred into an anaerobic jar. The growth medium was prepared according to Wu et al. [8]. The final fermentation liquid contained 10 mL growth medium, 9 mL fecal inoculum, and 100 mg EHPs. A blank control was set using the autoclaved ultrapure water, replacing the EHPs. All samples were incubated in anaerobic sealed tubes for 24 h at 37 °C.

#### 2.5.2. Determination of SCFA Concentration

The SCFA contents at the fermentation time of 12 h and 24 h were determined by gas chromatography (GC) using the Agilent 7890 series GC system equipped with an HP-FFAP column (30 m × 0.25 mm × 0.25 μm, Agilent Technologies, USA) [8].

#### 2.5.3. DNA Extraction and Sequence Analysis

After fermentation, the DNA of fermented samples was extracted using the E.Z.N.A.® Stool DNA Kit (Omega Bio-tek, Norcross, GA, USA). Next, the V3−V4 region of the bacterial 16S rRNA gene was PCR amplified with universal primers (338F and 806R) on a thermocycler PCR system (ABI GeneAmp 9700, Foster City, CA, USA). The PCR products were then purified using the AxyPrep DNA Gel Extraction Kit (Axygen Biosciences, Union City, CA, USA), and sequenced using the IlluminaHiSeq 2500 platform (San Diego, CA, USA) following the protocols developed/provided by MajorBio Bio-Pharm Technology Co., Ltd. (Shanghai, China).

### 2.6. Animal Studies

#### 2.6.1. Experimental Design

4-week-old male mice were purchased from GuangDong Medical Laboratory Animal Center (Guangdong, China). Experimental mice were housed under a standard feeding environment and supplied ad libitum food and pure water with free access for one week to adapt them to laboratory conditions. All experiments involving animals were conducted in accordance with the National Institutional Animal Care and Medical Ethics Committee of GuangDong Medical Laboratory Animal Center (C202301-5). Mice were administered with 3% dextran sulfate sodium (DSS) dissolved in drinking water for 7 consecutive days to induce colitis, followed by DSS replacement with drinking water for 3 days. To examine the effects of EHPs on DSS-induced colitis, the mice were divided into five groups (n = 10 per group): (1) Control group: water (2) DSS group: 3% DSS + water; (3) LEHPs: 3% DSS + 100 mg/kg EHPs; (4) MEHPs: 3% DSS + 200 mg/kg EHPs; (5) HEHPs: 3% DSS + 300 mg/kg EHPs. The EHPs were dissolved in water and administered by gavage feeding every day. The total experimental and EHP administration period was 10 days. The mice were weighed and monitored for stool consistency daily [19]. The mice colons were fixed in a 4% paraformaldehyde stationary solution for dehydration, embedded in paraffin, cut into 5 μm sections, and subjected to hematoxylin and eosin (H&E) staining for histopathological examinations [20].

#### 2.6.2. qRT-PCR

Total RNA from colitis tissue was extracted and purified using the Total RNA Extraction Kit (Foregen, Sichuan, China) and quantified by a Trace Ultraviolet-Visible Spectrophotometer. RNA was reverse transcribed to cDNA using the RNA PrimeScript™ RT Master Mix (Perfect Real Time, Takara Biomedical Technology Co., Ltd., Beijing, China). qRT-PCR was carried out using the SYBR Premix ExTaqTM (Takara). Fold changes from PCR analysis were determined using the ΔΔCT method [21]. The primers of RT-qPCR were listed in Table 1. 

### 2.7. Statistical Data Analysis 

All data are expressed as the mean ± standard deviation (SD), and the statistical analysis was carried out using SPSS 26.0 software (SPSS Inc., Chicago, IL, USA). The analysis methods included one-way analysis of variance (ANOVA) and Tukey’s test for two independent samples. 

## 3. Results 

### 3.1. Structural Characterization of EHPs

**Figure 2:**
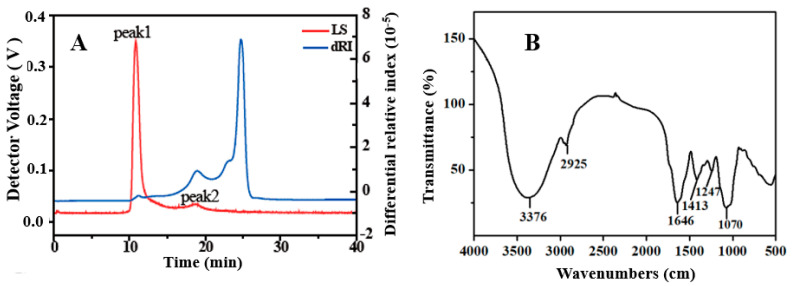
The molecular weight (**A**) and FITR spectrum (**B**) of EHPs.

EHPs were prepared through the conventional heating method, containing carbohydrate and protein contents of 75.30% and 2.02%, respectively (Table 2). This result revealed that EHPs comprised polysaccharide transport chains and protein residues or residual free proteins [22]. Moreover, EHPs consisted of galactose (Gal), glucose (Glu), glucuronic acid (GluA), arabinose (Ara), and mannose (Man) with the respective molar ratio percentages of 55.34:21.71:8.30:6.30:6.12, indicating Gal and Glu as the major monosaccharide components. The HPSEC chromatograms of EHPs exhibited two peaks (Figure 2A) at a ratio of 15.8:84.2, with the corresponding Mw values of 7.70 × 10^3^ kDa and 1.76 × 10^2^ kDa, further indicating that EHPs are heteropolysaccharides. In a previous report, three polysaccharides extracted from *Sagittaria sagittifolia* L. contained Glu and Gal with varying molar percentages and were classified as heteropolysaccharides by Gu et al. [22]. 

The FTIR spectra were recorded for structural insights into EHPs (Figure 2B). A broad absorption peak at 3376 cm^−1^ could be attributed to the O-H stretching vibration and the rise at 2925 cm^−1^ to the C-H stretching vibration. The absorption peaks at 1646 cm^−1^ and 1413 cm^−1^ originated from the asymmetric and symmetric vibrations of the COO^-^ group, indicating the presence of uronic acid [22], which is consistent with the monosaccharide composition of EHPs. The absorption peak of the methyl-ester around 1700 cm^−1^ was not observed, while the C-O stretching of the acetyl group can be seen at 1247 cm^−1^. These results indicate that EHPs are a type of acetylated and non-methyl-esterified polysaccharides [23]. In addition, the peak at 1070 cm^−1^ suggests the presence of pyranosyl glycoside bonds in the EHPs. 

### 3.2. In Vitro Absorption Characteristics of Polysaccharides 

**Figure 3:**
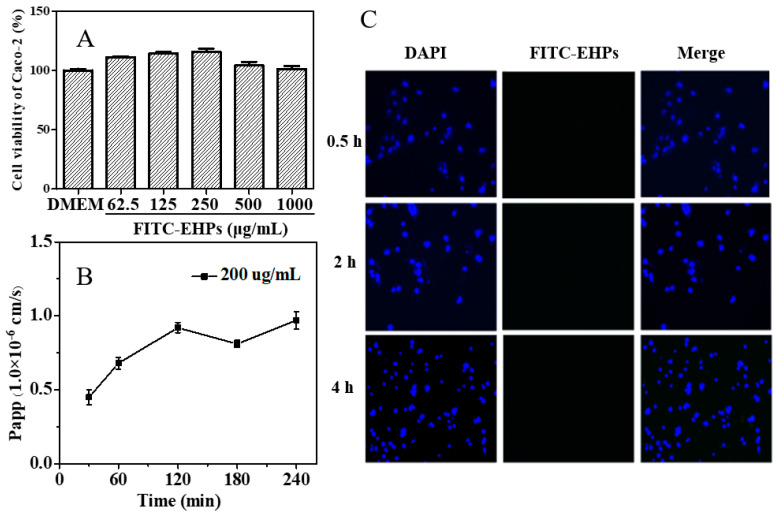
Toxicity of FITC-EHPs on Caco-2 cells (**A**), Papp value (**B**), and uptake of FITC-EHPs in Caoco-2 cells (**C**).

Caco-2 cells can slowly differentiate into a monolayer exhibiting many properties similar to the small intestinal villus epithelium and, thus, have been used to study the mechanism of nutrient absorption and transport [24,25]. To explore the intestinal absorption of EHPs by Caco-2 cell monolayers, the cytotoxic effect of FTIC-EHPs on Caco-2 cells was first examined by the CCK-8 assay (Figure 3A). The Caco-2 cell viability exceeded 100%, even when the concentration of FTIC-EHPs reached 1000 μg/mL, suggesting that FTIC-EHPs had no toxicity effects on Caco-2 cells. Furthermore, Caco-2 cells were grown on a polycarbonate film for 21 days to obtain the monolayers to study EHP transport. The TEER value is a strong indicator of the epithelial barrier integrity of cell monolayers; a high TEER value signifies tight junctions between the cell monolayers [24,25]. In this study, the TEER values of monolayers exceeded 500 Ω·cm^2^ after 21 days of culture, satisfying the experimental requirement. 

The paracellular and transcellular pathways are the two main pathways reported for macromolecule transport through the intestinal epithelial layer [26,27]. The paracellular pathway is restricted due to the tight junctions; typically, the gaps between the epithelial cells do not allow the passage of high Mw polysaccharides (Mw > 3.5 kDa) [28]. To support the smooth passing of these polysaccharides, the tight junctions must be relaxed or open; however, this may cause a decrease in the TEER value [24,25]. In this study, no apparent drop in TEER value was observed at any time, indicating the probable transport of EHPs across the Caco-2 cell monolayers through the transcellular pathway. The transport process of this pathway is mediated by crossing both the apical and basolateral membranes through passive diffusion or carrier vesicle-mediated processes.

The Caco-2 cell monolayer-associated Papp value is closely related to the intestinal absorption ability of a substance [29]. Generally, a Papp value higher than 1.0 × 10^−6^ cm/s denotes ~70–100% absorptivity of a drug. On the contrary, if the Papp value is lower than 1.0 × 10^−6^ cm/s, the drug can be regarded as poorly absorbed (~0–20%) [18,25]. The Papp values of FITC-EHPs detected at various time points within 4 h were observed in the range of 0.45 × 10^−6^ cm/s to 0.97 × 10^−6^ cm/s (Figure 3B), suggesting that FITC-EHPs are poorly-absorbed biological macromolecules. The cellular uptake of FITC-EHPs further confirmed this result, as shown in Figure 3C. A slight green fluorescence was observed in Caco-2 cells, even after incubation with a high concentration of FITC-EHPs (1000 ug/mL), indicating the uptake of only a few EHP molecules. Consequently, the unabsorbed EHPs could enter the colon as a source of carbohydrates and be fermented by the intestinal microbiota to regulate colon health.

### 3.3. In Vitro Fermentation of EHPs

#### 3.3.1. Changes in Intestinal Microecology

**Figure 4:**
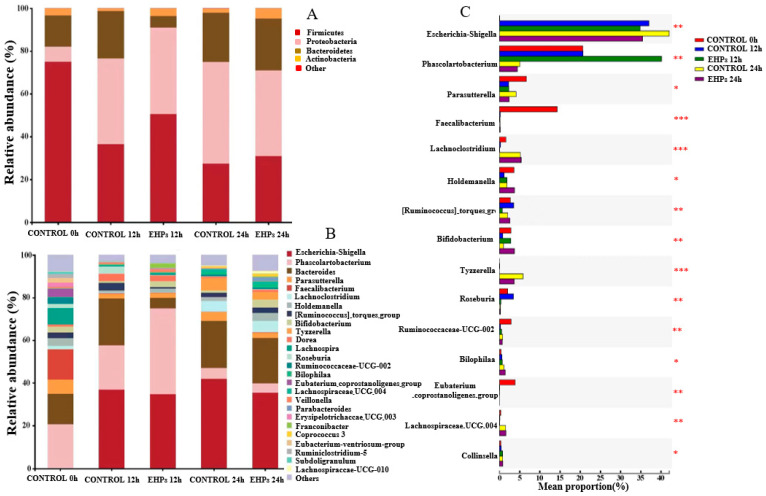
Relative abundance levels of the gut microbial community at the phylum (**A**), genus (**B**), and variance analysis of genus level (**C**). * *p* < 0.05, ** *p* < 0.01, *** *p* < 0.001 versus the control group.

The effect of EHPs on intestinal microbiota composition was investigated by in vitro fermentation followed by bacterial 16S rRNA sequencing. The three fermentation samples were analyzed at the phylum level to determine microbiota composition (Figure 4A). *Firmicutes*, *Bacteroidetes*, *Actinobacteria,* and *Proteobacteria* were observed as the major bacterial phyla, while other microflora accounted for only 0.01–0.03%, which is consistent with previous reports [8,16,30]. Compared with the control 24 h group, the relative abundance of *Firmicutes* and *Actinobacteria* increased, and that of *Proteobacteria* decreased in the EHP-supplemented fermentation groups, suggesting that the presence of EHPs significantly boosted the growth of *Firmicutes* and *Actinobacteria*, while inhibiting *Proteobacteria*. Generally, acidic conditions can stimulate *Firmicutes* growth during fecal fermentation [8]. *Firmicutes* are dominant intestinal flora that can ferment dietary indigestible carbohydrates into SCFAs, thus promoting host health [31]. *Actinobacteria* are pivotal in the maintenance of gut homeostasis and are widely used as probiotics, especially *Bifidobacteria* [30,31]. Although *Proteobacteria* is the most abundant phylum in healthy humans, it comprises some well-known opportunistic pathogens, such as *Escherichia-Shigella, enterohepatic Helicobacter* species, and *Campylobacter concisus*, which may cause intestinal flora imbalance, inflammation, and even chronic colitis [31].

At the genus level (Figure 4B,C), the relative abundance of *Bifidobacterium* (0.91% vs. 3.86%) and *Holdemanella* (1.81% vs. 3.64%) enhanced markedly with EHP intervention compared with the control 24 h group. *Lachnospiraceae* UCG 004 (0.14% vs. 1.12%) also presented increasing trends, though not significantly. In addition, an evident decreasing tendency was observed for *Escherichia-Shigella* (42.08% vs. 35.46%), *Tyzzerella* (5.91% vs. 3.54%), and *Parasutterella* (4.09% vs. 2.39%). Notably, *Bifidobacterium* is recognized as a key member of the gut microbiota and has been used as a commercial probiotic. In addition, *Bifidobacterium* can produce various glycosidases to hydrolyze macromolecular carbohydrates, such as EHPs [8]. *Escherichia-Shigella* can utilize low Mw carbon sources to maintain growth but cannot feed on polysaccharides due to the lack of carbohydrate-active enzymes [8]. Hence, the relative abundance of *Bifidobacterium* increased, and that of *Escherichia-Shigella* decreased in EHP-containing groups. These results indicate that EHPs could regulate the composition of intestinal microflora by stimulating the growth of beneficial microbiota and inhibiting potential pathogens. 

#### 3.3.2. Changes in SCFA Content

SCFAs are primary metabolites of dietary carbohydrates, which have been shown to exert multiple favorable effects on health maintenance [32,33]. In this study, the type and concentration of SCFAs were determined in the fermentation broth after an interval of 4, 8, 12, and 24 h (Table 3). The difference in total SCFA concentration between EHP-supplemented and control groups was insignificant during the early fermentation stage. However, after 24 h of fermentation, the concentration of total SCFAs in the EHP-supplemented group (32.75 ± 1.10 mmol/L) was remarkably higher than that in the control group (26.46 ± 2.55 mmol/L). The main types of SCFAs were found to be acetic acid, propionic acid, and butyric acid. Acetic acid content reached 8.82 ± 0.32 mmol/L in the EHP-supplemented groups at 24 h, which was moderately higher compared with the control group (6.10 ± 0.46 mmol/L). Being the major fermentation product of *Bacteroidetes*, acetic acid plays a crucial role in the metabolism of carbohydrates and fats [34]. Furthermore, the concentration of propionic acid was also elevated in the EHP-supplemented groups (9.92 ± 0.98 mmol/L) than in the blank group (7.01 ± 0.30 mmol/L). There is a good correlation between propionic acid and intestinal immune cells because propionic acid can alleviate intestinal immune stress and promote intestinal environmental homeostasis [16]. Likewise, the valeric acid concentration increased in groups with EHPs (0.90 ± 0.05 mmol/L) compared to that in the control group (0.41 ± 0.02 mmol/L). Valeric acid has been reported to promote the growth of intestinal epithelium with a beneficial effect on colitis. At the genus level, the concentration of valeric acid positively correlated with *Lachnospiraceae* [35]. However, the present data revealed no considerable change in the concentrations of butyric, isobutyric, and isovaleric acids between the EHP-supplemented and control groups during the entire fermentation period. Notably, the contents of acetic and propionic acids in the EHP-containing group were higher than those in the control group at different fermentation stages, which is in accordance with previous studies [36]. Thus, EHPs show a good potential to produce more SCFAs and regulate host health. The potential protective effects of EHP supplementation on digestive tract diseases were further confirmed using DSS-induced UC mouse models.

### 3.4. Therapeutic Effects of EHP Treatment in DSS-Induced Colitis Mice

#### 3.4.1. EHP Supplementation Ameliorated Colitis Symptoms

**Figure 5:**
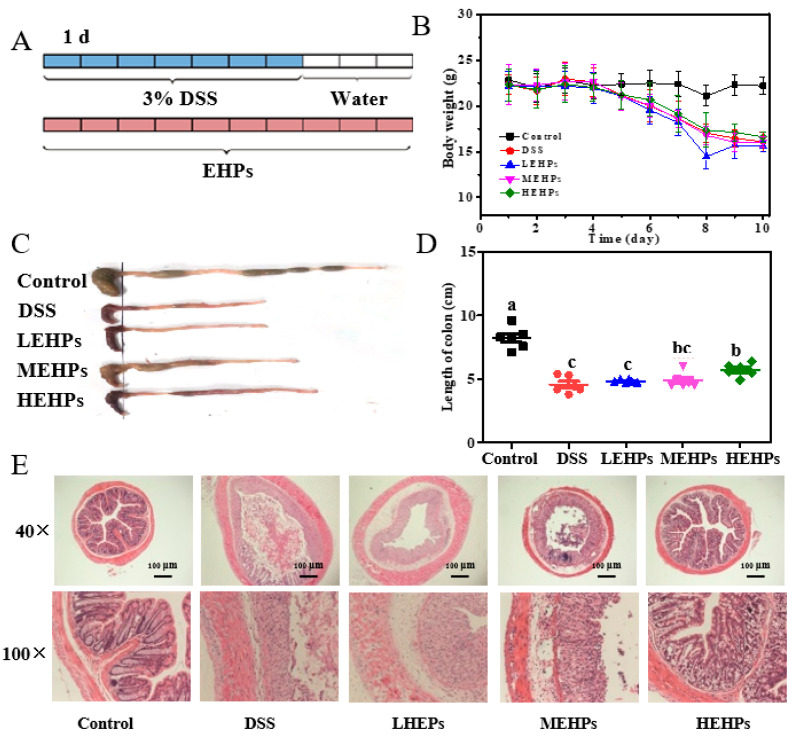
Therapeutic effects of EHPs on DSS-induced colitis. The mice were supplied with water or 3% DSS in drinking water for 10 d (**A**). Body weight loss during the DSS treatment (**B**). Images of mouse colons of different groups (**C**). Histogram statistics of colon length (**D**). H&E staining of colonic sections (40×, 100×) (**E**). The letters (a–c) indicate the significant differences of all treatment groups (*p* < 0.05).

DSS-induced acute colitis could directly destroy the integrity of the colonic epithelial barrier, causing infiltration of inflammatory cells and intestinal microenvironment imbalance [37]. In this study, the effect of EHPs on intestinal functions was investigated in a DSS-induced colitis mouse model and the intervention schedule is shown in Figure 5A. The DSS model shows similar symptoms of human UC, such as body weight loss, diarrhea, and rectal bleeding [37]. The EHP treatment alleviated these adverse changes (Figure 5B–D). Especially, the colon length in the HEHPs group increased (6.15 cm), with a significant difference from the DSS group (Figure 5C,D). These results confirmed the successful establishment of the UC model and alleviation of the typical colon atrophy induced by DSS after EHP treatment.

For further verification, H&E staining of the colon was adopted as the most direct method to assess the extent of colonic injuries. The crypt deformation, goblet cell disappearance, epithelial damage, and inflammatory cell infiltration were significantly alleviated in EHP-supplemented groups, especially in the HEHPs group, compared to that in the DSS group (Figure 5E). Thus, treatment with EHPs significantly reversed the colon tissue damage (*p* < 0.01).

#### 3.4.2. EHP Treatment Inhibited Colonic Inflammation

**Figure 6:**
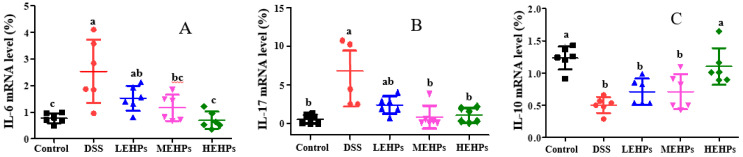
Effect of EHPs on the pro-inflammatory and anti-inflammatory cytokines in colonic tissue. (**A**,**B**) Pro-inflammatory cytokines (IL-6 and IL-17) in colonic tissue. (**C**) Anti-inflammatory cytokines (IL-10) in colonic tissue. The letters (a–c) indicate the significant differences of all treatment groups (*p* < 0.05).

The imbalance between pro-inflammatory and anti-inflammatory factors plays an important role in UC pathogenesis [38]. Accumulating evidence showed IL-6 and IL-17 as the primary cytokines in experimental colitis, and the average numbers of IL-6 and IL-17 were significantly increased in UC patients [37]. In inflammatory bowel disease, the anti-inflammatory cytokine IL-10 is pivotal for controlling the inflammatory responses to enteric organisms. Additionally, higher IL-10 levels have been shown to provide maximum protection against in vivo colitis [39]. Compared with the control group, the mRNA expression of inflammatory mediators (IL-6, IL-17) was remarkably elevated in the DSS group. However, EHP treatment significantly downregulated the expression levels of IL-6 and IL-17, particularly in the HEHPs group (Figure 6A,B). The expression of IL-10 was dramatically declined in the DSS-induced mice, while EHP treatment upregulated IL-10 levels (Figure 6C). Therefore, EHP supplementation inhibited the UC-associated pathological inflammation by reducing the expressions levels of pro-inflammatory factors and enhancing those of anti-inflammatory factors.

## 4. Discussion

UC is a refractory chronic bowel disease gradually increasing worldwide. Current treatment options, including corticosteroids, aminosalicylate, immunomodulators, and monoclonal antibodies, often lack clinical efficacy with multiple harmful side effects [37]. Therefore, the development of new treatment methods and management strategies to treat patients according to UC severity is necessary. Numerous studies have shown that dietary polysaccharides could reduce UC-associated inflammation and symptoms [40]. In this study, the in vitro utilization of EHPs by intestinal microbiota could modulate microbiota growth. Furthermore, EHP supplementation showed beneficial effects on DSS-induced UC mice, alleviating body weight loss and colon shortening and suppressing the inflammatory responses in the colon. 

The efficacy of *Bifidobacterium* in UC treatment has been reported previously [39,40,41]. *Bifidobacterium* strains proved beneficial in inducing and maintaining UC remission, exhibiting regulatory activities that contributed to controlling intestinal inflammation. In this study, EHP treatment increased the relative abundance of *Bifidobacterium*, which could reduce the inflammatory reaction associated with UC. In inflammatory bowel disease gut microbiota, the number of *Proteobacteria* was significantly elevated, especially *Escherichia-Shigella*, which has been considered a signature characteristic of dysbiosis in gut microbiota [42]. Thus, lower levels of *Proteobacteria* are likely to be beneficial to host health. Here, the relative abundance of *Proteobacteria* at the phylum level and *Escherichia-Shigella* at the genus level markedly declined after EHP fermentation. These findings indicate that EHPs might exhibit a therapeutic potential against UC, mediated by promoting the growth of *Bifidobacterium* and suppressing proteobacterial growth, especially *Escherichia-Shigella*. Overall, the effectiveness of EHP treatment in UC patients has been demonstrated.

## 5. Conclusions

This study presented the absorption and fermentation characteristics of EHPs. The indigestible and poorly absorbable EHPs were degraded by intestinal microbiota, promoting the production of acetic, propionic, and valeric acids. Additionally, EHP fermentation altered the diversity of intestinal microbiota, significantly increasing the relative abundance of *Bifidobacteria* and reducing that of *Escherichia-Shigella* at the genus level. Moreover, EHP treatment effectively suppressed the colitis symptoms in mouse models, which manifested as improvements in body weight loss, increasing colon length, reducing inflammatory cell infiltration and restoring intestinal epithelial barrier integrity. The current study suggests that EHP supplementation could be a promising nutritional therapeutic strategy for inflammatory bowel diseases. However, the current research has a limitation of not collecting and analyzing the feces of DSS-induced mice. In the future, the study will focus on the diversity, composition, and metabolites of microbiota in mouse colonic feces, to reveal the causality between intestinal microbiome changes and UC development during EHP treatment.

## Figures and Tables

**Table 1:** Primer Sequences for RT-PCR.

Genes	Forward Primer	Reverse Primer
*IL-6*	5′-AGCGATGATGCACTGTCAGA-3′	5′-GGAACTCCAGAAGACCAGAGC-3′
*IL-17*	5′-TTCTTTCAAACAAAGGACCAGC-3′	5′-GCAACCCAAGTAACCCTTAAAG-3′
*IL-10*	5′-GACTTCACCATGGAACCCGT-3′	5′-GGAGACTGCCCATTCTCGAC-3′

**Table 2:** Chemical properties and monosaccharide composition of EHPs.

Items	Content
Composition (w%)	
Carbohydrate	75.30 ± 0.04
Protein	2.02 ± 0.41
Monosaccharide composition (molar ratio %)	
Mannose (Man)	6.12
Rhamnose (Rha)	1.47
Glucuronic acid (GluA)	8.30
Galacturonic acid (GalA)	0.77
Glucose (Glu)	21.71
Galactose (Gal)	55.34
Arabinose (Ara)	6.30

**Table 3:** Production changes of SCFAs during fermentation in vitro.

Sample	Time (h)	SCFAs(mmol/L)						
		Acetic Acid	Propionic Acid	Butyric Acid	Isobutyric Acid	Valeric Acid	Isovaleric Acid	Total
Control	0	ND	ND	ND	ND	ND	ND	ND
	4	1.21 ± 0.21 ^f^	1.51 ± 0.17 ^d^	0.18 ± 0.01 ^e^	0.07 ± 0.00 ^c^	0.07 ± 0.00 ^d^	0.05 ± 0.00 ^b^	3.09 ± 0.11 ^f^
	8	2.61 ± 0.09 ^e^	2.43 ± 0.29 ^cd^	1.53 ± 0.16 ^cd^	0.10 ± 0.01 ^c^	0.11 ± 0.01 ^cd^	0.05 ± 0.00 ^b^	6.63 ± 0.10 ^de^
	12	5.62 ± 0.26 ^c^	6.20 ± 0.35 ^b^	5.80 ± 0.67 ^b^	1.70 ± 0.18 ^b^	0.25 ± 0.01 ^bc^	0.16 ± 0.01 ^b^	19.73 ± 1.84 ^c^
	24	6.10 ± 0.46 ^c^	7.01 ± 0.30 ^b^	7.34 ± 0.27 ^a^	3.46 ± 0.39 ^a^	0.41 ± 0.02 ^b^	2.14 ± 0.12 ^a^	26.46 ± 2.55 ^b^
EHPs	0	ND	ND	ND	ND	ND	ND	ND
	4	1.64 ± 0.08 ^ef^	1.85 ± 0.09 ^d^	0.24 ± 0.01 ^de^	0.07 ± 0.01 ^c^	0.06 ± 0.00 ^d^	0.04 ± 0.00 ^b^	3.90 ± 0.67 ^ef^
	8	3.70 ± 0.42 ^d^	3.48 ± 0.35 ^c^	2.12 ± 0.42 ^c^	0.13 ± 0.01 ^c^	0.14 ± 0.01 ^cd^	0.07 ± 0.00 ^b^	9.64 ± 0.35 ^d^
	12	7. 50 ± 0.50 ^b^	7.20 ± 0.24 ^b^	5.43 ± 0.46 ^b^	1.81 ± 0.17 ^b^	0.35 ± 0.01 ^bc^	0.22 ± 0.02 ^b^	22.51 ± 1.81 ^c^
	24	8.82 ± 0.32 ^a^	9.92 ± 0.98 ^a^	7.62 ± 0.39 ^a^	3.24 ± 0.40 ^a^	0.90 ± 0.05 ^a^	2.25 ± 0.19 ^a^	32.75 ± 1.10 ^a^

Values are expressed as mean ± SD (n = 3). Different letters indicate significant differences under different fermentation conditions; ND: not detected.

## Data Availability

Data is contained within the article.

## Abbreviations

Papp	Apparent permeability coefficient
Ara	Arabinose
DSS	Dextran sulfate sodium
EHPs	*Euphorbia humifusa*-derived polysaccharides
FTIR	Fourier transform infrared spectroscopy
FITC	Fluorescein isothiocyanate
HPSEC	High-performance size exclusion chromatography
H&E	Hematoxylin-eosin
Gal	Galactose
Glu	Glucose
GluA	Glucuronic acid
Man	Mannose
SCFAs	Short-chain fatty acids
TEER	Transepithelial electrical resistance
UC	Ulcerative colitis
